# Antagonistic Activity of Fungal Strains against *Fusarium* Crown Rot

**DOI:** 10.3390/plants11030255

**Published:** 2022-01-19

**Authors:** Xingli Zhao, Dianyun Hou, Jiaqi Xu, Kaixuan Wang, Zhenjie Hu

**Affiliations:** 1 College of Agriculture, Henan University of Science and Technology, Luoyang 471000, China; hellen1984@haust.edu.cn (X.Z.); dianyun518@163.com (D.H.); hnkjdxxjq@163.com (J.X.); 18272862598@139.com (K.W.); 2 College of Horticulture and Plant Protection, Henan University of Science and Technology, Luoyang 471000, China

**Keywords:** antagonistic activity, identification, *Fusarium* crown rot, biocontrol gene

## Abstract

The crown rot of wheat is a destructive soil-borne pathogen that severely reduces the yield and quality of wheat. This study aimed to screen and identify the antagonistic strains against *Fusarium pseudograminearum* (*Fp*), which is the dominant pathogen associated with the crown rot of wheat in China, and evaluate their biosynthetic potential. The antagonistic strains were screened via a dual-culture antagonism assay, and then identified by combining the morphological characteristics and internal transcribed spacer gene sequencing. The polyketide synthases (PKS-I and PKS-II) and non-ribosomal peptide synthetase (NRPS) genes in the antagonistic strains were detected via specific amplification of chromosomal DNA. Eleven out of 157 fungal strains, including six strains with matrix competition and five strains with antibiosis, were obtained. The eleven antagonistic strains belonged to the following four genera: *Alternaria*, *Botryosphaeria*, *Phoma* and *Talaromyces*. The inhibition rate of six strains with matrix competition was greater than 50%, with *B. dothidea* S2-22 demonstrating the highest at 80.3%. The width of the inhibition zone of *T. trachyspermus* R-17 among the five strains with antibiosis was the widest at 11 mm. Among the eleven antagonistic strains, three strains of *A. alternata* and the strain *P. moricola* only contained the PKS-II gene, the strain *A. tenuissima* contained PKS-I and PKS-II genes, three strains of *B. dothidea* contained PKS-II and NRPS genes, while three strains of *T. trachyspermus* did not contain any genes. These results demonstrated potential strains for the biocontrol of the crown rot of wheat. In particular, *T. trachyspermus* R-17 can be investigated further as a promising agent, and the active substances secreted by antagonistic strains may be synthesized by other pathways.

## 1. Introduction

Wheat (*Triticum aestivum*) is an important cereal crop worldwide. Wheat planting areas in China cover 21 million ha and produce 19% of the total world production at 87.79 million tons of wheat every year [1]. However, due to the threat of various pests and diseases, the yield of wheat has been severely reduced. The crown rot of wheat is an economically important wheat disease commonly found in wheat-cultivating regions around the world that causes not only yield reduction, but also grain quality failure [2]. The yield in North China Plain, a major winter wheat-producing area, is remarkably reduced by the crown rot of wheat [3]. The crown rot of wheat is caused by several complex species of *Fusarium*, including *F. pseudograminearum (Fp*), *F. culmorum*, and *F. graminearum* [4]. However, *Fp* and *F. culmorum* are more pathogenic than *F. graminearum* and result in greater yield loss [5]. *Fp* is the dominant pathogen associated with the crown rot of wheat in the Pacific Northwest of the United States, while *F. culmorum* is the dominant species in eastern Victoria and South Australia, and is more prevalent than *Fp* in drier soils [6,7]. Four consecutive years of systematic investigation on the distribution and diversity of pathogens associated with the crown rot of wheat showed that *Fp* is the dominant species in the Huanghuai wheat-growing region of China [8].

*Fp* is a destructive soil-borne pathogen that can induce seedling blight, brown discoloration of lower stems, and whiteheads at the filling stage by infecting the wheat root and crown [9]. This pathogen may lead to contamination of wheat stubble grains and produce a wide variety of mycotoxins, such as deoxynivalenol and trichothecenes nivalenol, which are harmful to humans and animals [10]. Chemical fungicides extensively used in agroecosystems for plant disease management can cause a series of environmental pollution and health problems. Furthermore, the increasing number of fungal pathogens intensifies the resistance to many active ingredients of chemicals [11]. Therefore, biological control is an eco-friendly approach that has been used as an alternative to minimize the use of chemical pesticides. The use of microbial antagonists and their active metabolites is an effective strategy to control phytopathogenic fungi in sustainable agriculture [12]. However, the crown of wheat has attracted less research attention worldwide compared to other important fungal diseases of wheat, such as wheat rust and take-all, due to geographical limitations [13].

Previous studies isolated 157 endophytic fungi from the medicinal plant *Cornus officinalis* Sieb. et Zucc. The present study aims to determine the activity of these 157 strains against *Fp* via dual-culture plate antagonism, identify the strain with antifungal activity that combines morphological characteristics and internal transcribed spacer (ITS) sequence analysis, and analyze their biosynthetic potential through screening the functional genes PKS-I, PKS-II, and NRPS. The results can provide potential biocontrol strains for the control of the crown rot of wheat.

## 2. Results

### 2.1. Antifungal Activity of Testing Fungal Isolates

**Figure 1:**
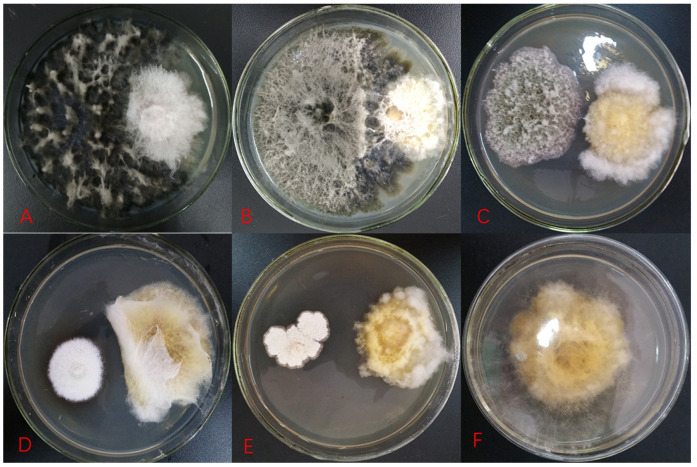
Antagonism of representative strains on *Fusarium pseudograminearum* after five days of confrontation culture. (**A**–**E**) represent strains L-02, S2-22, S1-03, R-35, and R-17 in order; wherein (**A**,**B**) are matrix competition and (**C**–**E**) are antibiosis; (**F**) is a control, *Fusarium pseudograminearum*.

**Figure 2:**
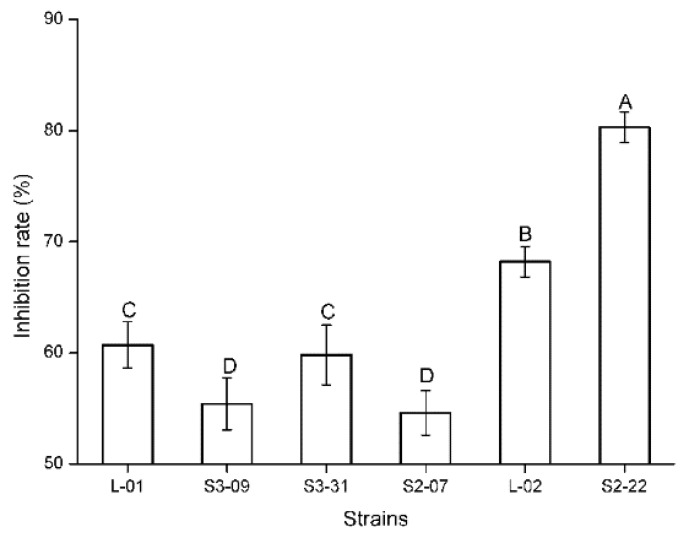
Inhibition rate of six strains against *Fusarium pseudograminearum* after five days of confrontation culture (Kruskal–Wallis test: *H* = 15.643, *p* = 0.008). The data in the figure are “mean ± SD”. Different letters indicate significant differences (α = 0.05).

**Figure 3:**
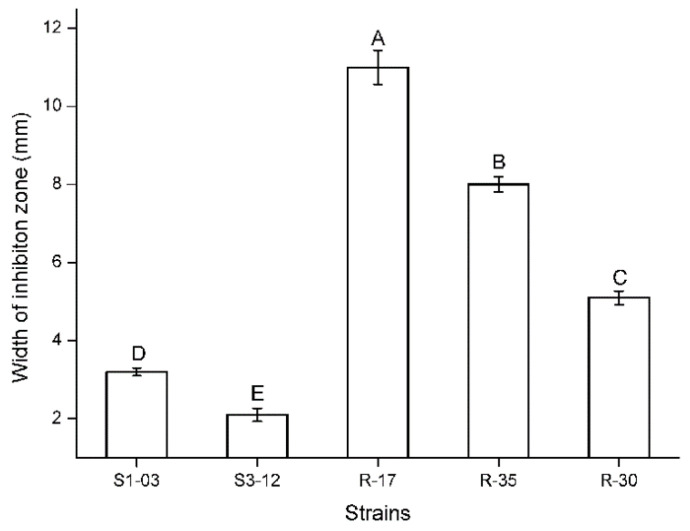
Width of inhibition zone of five strains against *Fusarium pseudograminearum* after five days of confrontation culture (ANOVA: *F* = 659.85, *p* < 0.001). The data in the figure are “mean ± SD”. Different letters indicate significant differences (α = 0.05).

The inhibition effect of antagonistic fungi on target strains in the dual-culture plate antagonism assay is mainly manifested in three types, namely, matrix competition, antibiosis, and mycoparasitism. In the present study, there were only matrix competition and antibiosis (Figure 1). Specifically, 152 strains had a competition effect and 5 strains had antibiosis. Competition is inevitable, and two fungi in co-culture can obstruct each other’s growth by competing for water, nutrients, and living space. Fast-growing fungi generally exhibit clear advantages in competition. However, the strain will be of further developmental value when the inhibition rate is more than 50%. The current study demonstrated that six fungi against *Fp* obtain an inhibition rate greater than 50% (55.4–80.3%) (Table 1, Figure 1). The statistical analysis indicated that the inhibition rates of these six strains against *Fp* were significantly different (Kruskal–Wallis test: *H* = 15.643, *p* = 0.008; Figure 2). Among them, the inhibition rate of strain S2-22 was the highest (Figure 1B). Antibiosis is mainly determined through metabolic products secreted by antagonistic strains to inhibit the growth of pathogenic fungi, and results in the emergence of inhibition zones around their biomass. Moreover, antibiosis is not necessarily related to the growth rate of the antagonistic strain. Five strains in the present study produced clear and distinct inhibition zones ranging from 2 mm to 11 mm after 5–10 d of confrontation culture (Table 1, Figure 1), in which the inhibition zone of strain R-17 was the widest (Figure 1E), and the statistical analysis indicated that the width of the inhibition zones of these five strains against *Fp* were also significantly different (ANOVA: *F* = 659.85, *p* < 0.001; Figure 3).

### 2.2. Identification of Isolates with Antifungal Activity

Eleven strains with antifungal activity were screened via an antagonism assay, and further identified on the basis of colony and spore characteristics and ITS (ITS1, intervening 5.8S and ITS2) phylogenetic analysis. The ITS sequence lengths of the antagonistic strains were in the approximate range of 500–600 bp. The similarities between the antagonistic strains and reference sequences in the NCBI database (http://www.ncbi.nlm.nih.gov/ (accessed on 11 January 2018)) were all above 99%. Detailed descriptions of the antagonistic strains, with their respective codes, GenBank accession numbers, and similar sequence homologs, are summarized in Table 1.

**Figure 4:**
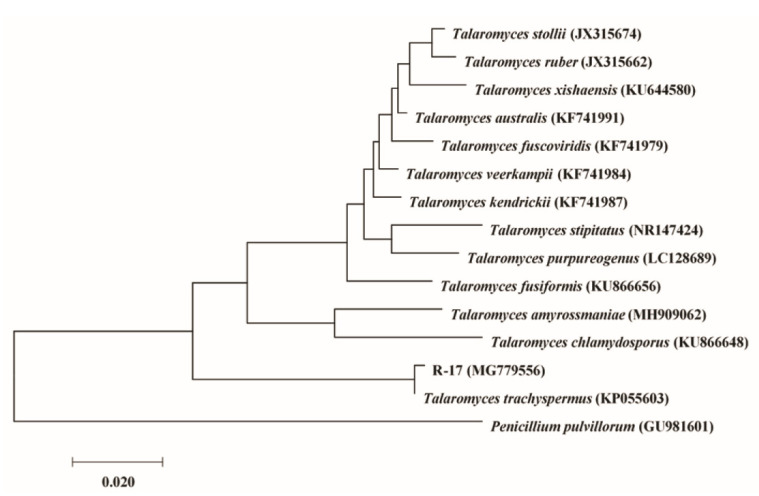
The phylogenetic tree of *Talaromyces trachyspermus* R-17 based on ITS sequence. The contents in parentheses represent the accession numbers in GenBank.

The six strains with matrix competition (Table 1) belonged to the genera *Alternaria* and *Botryosphaeria*. *Alternaria* included three isolates of *A. alternata* and one isolate of *A. tenuissima,* while *Botryosphaeria* only had two isolates of *B. dothidea*. The inhibition rates of *B. dothidea*, *A. alternata*, and *A. tenuissima* ranged from high to low, and the inhibition rate of two isolates of *B. dothidea* was higher than the values of the genus *Alternaria*. Five antibiotic strains (Table 1) belonged to the genera *Botryosphaeria*, *Phoma*, and *Talaromyces*. Among them, *Talaromyces* had three isolates of *T. trachyspermus*, and the two other genera each contained one isolate, namely, *B. dothidea* and *P. moricola*. Three isolates of *T. trachyspermus* demonstrated higher antibiosis than *B. dothidea* and *P. moricola*. Given that the *T. trachyspermus* strain R-17, with the strongest antibiosis, had the greatest potential for further development, it was used as an example, and a phylogenetic tree was constructed in detail, which showed the relationship between strain R-17 and other closer species belonging to the genus *Talaromyces;* and *Penicillium pulvillorum* was an outgroup (Figure 4).

### 2.3. Detection of PKS-I, PKS-II, and NRPS Gene Sequences

Eleven antagonistic strains exhibiting matrix competition or antibiosis to *Fp* were selected for the detection of polyketide synthase I and II (PKS-I and PKS-II), and nonribosomal peptide synthase (NRPS) genes (Table 1). Three strains of *A. alternata* and the strain *P. moricola* only contained the PKS-II gene. The strain *A. tenuissima* contained PKS-I and PKS-II genes. Three strains of *B. dothidea*, regardless of whether they showed competition or antibiosis, contained PKS-II and NRPS genes. However, these genes were absent in the three strains of *T. trachyspermus* exhibiting the maximum antibiosis.

## 3. Discussion

Studies on the biological control of the crown rot of wheat are relatively scattered because this disease can be caused by many pathogenic fungi and its distribution has geographical limitations. According to the investigation on different types of antagonistic fungi, including *F. equiseti*, *F. nygamai*, *Trichoderma harzianum*, and *A. infectoria*, and the use of different forms of nitrogen sources in the displacement of *Fp* from infected stubble pieces, the combination of *T. harzianum* and nitrogen can effectively increase the displacement from previously infected stubbles to control *Fp* [14]. The use of fungicide seed treatment, cultivar resistance, and systemic acquired resistance induced by *Bacillus mycoides* is an integrated approach to reduce damp off and protect wheat against *Fp* throughout the growing season. However, no significant difference was found between the use of the integrated approach and application of each control strategy individually in reducing CR severity under glasshouse conditions [15].

The obtained biocontrol strain R-17 in this study showed not only significant antibiosis to *Fp,* but also to *F. graminae*, which was isolated from rice, and verified through other experiments in our research group. Therefore, the strain R-17 may have antagonistic effects on multiple *Fusarium* spp., and may cause crown rot of wheat. In addition, the crown rot, sheath blight, and take-all of wheat are three of the most important soil-borne wheat diseases worldwide. The biological activity of the strain R-17 against wheat sheath blight and take-all was also verified via the dual-culture plate antagonism as soon as this biocontrol strain was screened. The results showed that the strain R-17 exhibited acceptable antibiosis against the two pathogenic fungi. Given that antibiosis can inhibit or kill harmful fungi via secondary metabolites secreted by the antagonistic fungi, the industrial production of active substances can be realized via scale-up fermentation of the strain. Based on this analysis, the antagonistic strain R-17 demonstrates satisfactory development potential as a fungicide for controlling soil-borne diseases in wheat.

The pathogenicity of pathogenic fungi is related to the pigment they produce [16]. Fungi typically rely on spores to produce toxic substances, and the synthesis of pigments is related not only to spore formation, but also to spore protection from damage by ultraviolet light [2,17]. The yellow pigment content of the pathogenic fungus *Fp* clearly decreased in the dual-culture plate (Figure 1C–E), and *Fp* produced a significantly reduced number of spores under microscopic observation compared with the control in this study. Pigments are an important virulence factor for the pathogenicity of some fungi [18]. The decrease in yellow pigment content of the pathogenic fungus *Fp* in this study was more evident on the side near the antagonistic strain R-17 than on the side away from the antagonist (Figure 1E). These findings demonstrated that active substances produced by the antagonistic strain R-17 can interfere with the pigment formation of pathogenic fungus *Fp*, further inhibit spore formation, and, thus, reduce the pathogenic virulence of *Fp*.

The genus *Talaromyces* belongs to the fungal species that produces asci in chains, and Benjamin described it as a sexual state of *Penicillium* [19]. On the basis of nuclear ribosomal ITS regions, small subunit nuclear ribosomal DNA, and/or large subunit ribosomal DNA, *Talaromyces* is recognized as a distinct genus from *Penicillium,* and species previously attributed to the *Penicillium* subgenus *Biverticillium* were transferred into *Talaromyces* according to phylogenetic analysis [20]. *Talaromyces* species are distributed worldwide and have been isolated from a wide range of resources, such as soil samples, food samples, marine sponges of coral reefs, coastal plant roots, and plant endophytes [21]. *Talaromyces* species can synthesize bioactive compounds that have promising applications in medicine, agriculture, and various industries, such as antibiotics, antimicrobials, or food dyes. Although altenusin and dehydroaltenusin are sphingomyelinase inhibitors that were originally metabolites of *Alternaria tenuis*, they can be produced in large amounts by *Talaromyces* species, and have anticancer, antifungal, and anti-*Trypanosoma* properties [22]. Altenusin and dehydroaltenusin show strong antifungal activity against *Aspergillus fumigatus* and *Candida albicans* [23].

*T. trachyspermus* is an important *Talaromyces* species of antagonistic fungi. The antibiosis of this fungus shows that its metabolite trachyspic acid can inhibit the tumor cell heparanase [24]. Recent studies have been conducted on the use of *T. trachyspermus* for biological control, with an improvement in people’s environmental awareness. The evaluation of the mycocidal activity of five marine-derived beneficial fungi against plant pathogenic fungi showed that the crude extract of ethyl acetate of *T. trachyspermus* can effectively inhibit the mycelial growth of *Alternaria brassicicola*, *Colletotrichum capsici*, *Helminthosporium maydis*, *Pythium aphanidermatum*, *Rhizoctonia solani*, and *Sclerotium rolfsii* [25]. One isolate of *T. trachyspermus* recovered from the necrotic tissue of broomrape (*Orobanche* spp.) in Iran caused a significant reduction in the number of tubercles, and the number and fresh weight of broomrape shoots (*P* = 0.01), thereby indicating its potential as an antagonist for the biocontrol of broomrape [26]. *T. trachyspermus* isolated from the medicinal plant *Withania somnifera* (winter cherry) exhibited high activity in hydrolytic enzymes, protease, chitinase, amylase, cellulase, and pectinase, which are required for antagonistic properties, and demonstrated satisfactory potential as a biocontrol agent against the plant pathogenic fungus *Sclerotinia sclerotiorum*. Meanwhile, this strain was characterized by a high level of indole acetic acid, siderophore synthesis, and phosphate solubilization activities important for plant growth promotion [27].

The presence of PKS and NRPS genes in antagonistic strains was screened to determine their correlation with antifungal activity [28]. PKS-I, PKS-II, and NRPS genes are involved in the synthesis of polyketides and nonribosomal peptides, with a remarkable range of bioactive substances related to anticancer, antiparasite, antifungal, and immunosuppression properties [29,30]. Some of the 11 strains showed satisfactory inhibitory activity against *F. pseudograminearum*, and contained two functional genes, while some only contained one, or even none, of the functional genes in the present study. The correlation between the three target genes and the antifungal activity of the strain is not always direct, which is consistent with previous reports [28,31]. The absence of three target genes in three antibiotic strains belonging to the genus *Talaromyces* revealed that bioactive substances are likely synthesized by other metabolic pathways [32]. The exploration of biocontrol genes related to antibiotic strains can be a research objective in a future investigation.

## 4. Materials and Methods

### 4.1. Strains

*F. pseudograminearum* was provided by the Plant Pathology Research Center of the College of Forestry, Henan University of Science and Technology, China. The 157 endophytic fungi were conserved by the Center for Evaluation and Utilization of Medicinal Plant Resources of the College of Agriculture, Henan University of Science and Technology, China. The hyphae or spores of these strains were stored in an aqueous glycerol solution (30%, *v*/*v*) at −80 °C. The fungal inoculum was prepared from these frozen stocks by transferring the hyphae or spores onto potato dextrose agar (PDA) media.

### 4.2. Determination of Antifungal Activity

The antifungal activity of the 157 endophytic fungi against *Fp* was determined by modifying the dual-culture plate antagonism described previously [33]. All fungi were cultured on PDA media at 26 °C for 7 d. Then, 5 mm plugs of each endophyte and *Fp* were co-cultured in 90 mm sterile Petri dishes, with approximately 28 mL of PDA yielding a final depth of 5 mm, and incubated at 26 ± 0.5 °C. The plugs were placed symmetrically on each side of the Petri dishes, 4 cm apart from each other. *Fp* alone was inoculated as the control. The growth radii of endophyte and *Fp* were recorded at regular intervals every day as well as the production of an inhibition zone, and this was not terminated until the *Fp* stopped growing or the size of the control colony was close to that of the plate. There were three replicates for each treatment, and the experiment was repeated three times.

Percent inhibition rate (IR) of mycelial growth was calculated as IR = (R1 − R2)/R1 × 100%, where R1 is the colony radial growth of *Fp* in control plates and R2 is the radial growth of fungi in tested plates.

The Kruskal–Wallis test was performed to analyze inhibition rate of strains on the *Fp*. The Bonferroni method was used for pairwise comparison. One-way analysis of variance (ANOVA) was employed to analyze width of inhibition zone of strains on the *Fp*. The normality test and homogeneity test of variances were carried out before analysis of covariance. The least significant difference (LSD) method was used for pairwise comparison.

### 4.3. Morphological Identification of Fungi with Antifungal Activity

The fungi with antifungal activity against *Fp* were cultured on PDA media at 26 °C for approximately 10 d to record their colony characteristics regularly every day, including color, texture, topography, border type, radial growth rate, the presence of aerial mycelia and substrate mycelia, and distinctive reverse colony color. Meanwhile, the presence of spores was monitored until the morphology and size of conidia were clearly observed under the light microscope. All the results obtained were compared with taxonomic keys [34].

### 4.4. DNA Extraction, PCR Amplification and ITS Sequencing

The strains with antifungal activity were subjected to DNA extraction, amplification, and sequencing of the ITS region of rRNA gene. The genomic DNA was extracted using the CTAB method [35]. Universal primers ITS1 (5′-TCCGTAGGTGAACCTGCGG-3′, forward) and ITS4 (5′-TCCTCCGCTTATTGATATGC-3′, reverse) were used for amplification of the ITS region, which consisted of ITS1, 5.8S and ITS2 regions of the rDNA [36]. PCR reaction [37] was performed in a total volume of 25 μL containing 12.5 μL of 2 × Taq PCR Green Mix, 0.5 μL of ITS1 (10 μM), 0.5 μL of ITS4 (10 μM), 1 μL of template DNA and 10.5 μL of sterile double-distilled water. The PCR cycling protocol [37] consisted of initial denaturation at 94 °C for 4 min and 35 cycles of denaturation, annealing and elongation at 94 °C for 30 s, 55 °C for 30 s, and 72 °C for 40 s, which was followed by a final elongation step of 72 °C for 10 min. As a negative control, the template DNA was replaced by sterile double-distilled water. After amplification, an aliquot was analyzed by running it on a 1% (*w*/*v*) TAE agarose gel stained with nucleic acid fuel Goldview, which was added at the volume ratio of 1/20 and visualized under UV light if a clear and bright band was present. The PCR products were compared to a molecular size standard 2 kb plus DNA ladder. All the molecular reagents were purchased from Sangon Biotech (Shanghai, China).

When the amplification product was detected as a single bright band, it was sent to Sangon Biotech (Shanghai, China) for sequencing. Then, the ITS sequences were matched with the nucleotide database using the Basic Local Alignment Search Tool (BLAST) of the US National Centre for Biotechnology Information (NCBI). The sequences were aligned using CLUSTAL W software packaged with MEGA 7.0 under default settings. When the similarity between a particular problem sequence and a phylogenetically associated reference sequence was ≥99%, the sequences were considered to be conspecific [38]. The phylogenetic tree was reconstructed and the evolutionary history was inferred using the neighbor-joining method. The robustness of the internal branches was also assessed with 1000 bootstrap replications. The evolutionary distances were computed using the maximum composite likelihood method and calculated in the units of the number of base substitutions per site.

Taxonomic assignment was based on similarity with reference sequences retrieved from GenBank and in consultation with observed colony and spore morphology. The ITS sequences of the screened fungal isolates were deposited in the NCBI GenBank and accession numbers were obtained.

### 4.5. Detection of PKS-I, PKS-II and NRPS Gene Sequences

The strains with antifungal activity were selected for detection of genes encoding polyketide synthases I and II (PKS-I and PKS-II) and nonribosomal peptide synthetases (NRPS). These three genes were amplified with the following three sets of primers: the PKS-I gene (1200−1400 bp) was amplified with K1F (5′-TSAAGTCSAACATCGGBCA-3′) and M6R (5′-CGCAGGTTSCSGTACCAGTA-3′) [39], the PKS-II gene (600 bp) was amplified with KSα (5′-TSGCSTGCTTGGAYGCSATC-3′) and KS_β_ (5′-TGGAANCCG CCGAABCCTCT-3′) [40], and the NRPS gene (700−800 bp) was amplified with A3F (5′-GCSTACSYSATSTACACSTCSGG-3′) and A7R (5′-SASGTCVCCSGTSCGGTAS-3′) [39].

The reaction was performed in a total volume of 25 μL containing 12.5 μL of 2 × Taq PCR Green Mix, 1 μL of each primer (10 μM), 1 μL of template DNA, 1.25 μL of dimethyl sulfoxide, and 8.25 μL of sterile double-distilled water. The DNA template was replaced by sterile double-distilled water in control reaction. The PCR cycling protocol consisted of initial denaturation at 94 °C for 5 min; 30 amplification cycles of 94 °C for 1 min; 57 °C for 1 min for amplification of the PKS-I and NRPS genes or 58 °C for 1 min for amplification of the PKS-II gene; 72 °C for 2 min; a final extension at 72 °C for 5 min (Metsa-Ketela et al. 1999; Ayuso-Sacido & Genilloud, 2005).

## 5. Conclusions

In this study, eleven antagonistic strains against *Fusarium* crown rot of wheat, including six strains with matrix competition and five strains with antibiosis, were obtained from 157 endophytic fungi. In particular, *Talaromyces trachyspermus* R-17 with antibiosis was determined as a promising agent for the biocontrol of crown rot of wheat. The detection of PKSs and NRPS genes in the eleven antagonist strains indicated that the active substance secreted by antagonistic strains may be synthesized by other pathways. The results showed potential strains for the biocontrol of *Fusarium* crown rot of wheat.

## Figures and Tables

**Table 1:** Antagonism, ITS analysis and detection of PKS/NRPS genes of eleven antagonistic strains against *Fusarium pseudograminearum*.

Strain Number	Antagonism	Most Closely Related Strain (Accession Number)	GenBank Accession Number	Maximum Identity %	Presence of Gene PKS-I PKS-II NRPS		
L-01	MC(60.7)	*Alternaria alternata* (MT134991)	MG779581	100	−	+	−
S3-09	MC(55.4)	*Alternaria alternata* (KJ526174)	MG779560	100	−	+	−
S3-31	MC(59.8)	*Alternaria alternata* (MG214868)	MG779590	100	−	+	−
S2-07	MC(54.6)	*Alternaria tenuissima* (MH790256)	MG779609	99	+	+	−
L-02	MC(68.2)	*Botryosphaeria dothidea* (KY788303)	MG779587	99	−	+	+
S2-22	MC(80.3)	*Botryosphaeria dothidea* (KF293775)	MG779603	99	−	+	+
S1-03	AB (3.2)	*Botryosphaeria dothidea* (KP183180)	MG779610	99	−	+	+
S3-12	AB (2.1)	*Phoma moricola* (KF293794)	MG779600	99	−	+	−
R-17	AB (11.0)	*Talaromyces trachyspermus* (KP055603)	MG779556	99	−	−	−
R-35	AB (8.0)	*Talaromyces trachyspermus* (KJ482651)	MG779553	99	−	−	−
R-30	AB (5.1)	*Talaromyces trachyspermus* (MK271298)	MG779552	99	−	−	−

Note 1: The letters in the antagonism represent different antagonistic effects. MC and AB represent matrix competition and antibiosis, respectively. 2: the numerals in parentheses after MC and AB represent “inhibition rate%” and “width of inhibition zone (mm)”, respectively, and all the data shown are the average values under different treatments. 3: the signs “+” and “−” represent the presence and absence of the corresponding gene, respectively.

## Data Availability

The data presented in this study are openly available in [repository name e.g., FigShare] at [doi], reference number [reference number]. See original data for details.
